# miR-371a-3p, miR-373-3p and miR-367-3p as Serum Biomarkers in Metastatic Testicular Germ Cell Cancers Before, During and After Chemotherapy

**DOI:** 10.3390/cells8101221

**Published:** 2019-10-08

**Authors:** Ximena Rosas Plaza, Ton van Agthoven, Coby Meijer, Marcel A. T. M. van Vugt, Steven de Jong, Jourik A. Gietema, Leendert H. J. Looijenga

**Affiliations:** 1 Department of Medical Oncology, University Medical Center Groningen, University of Groningen, NL-9713 GZ Groningen, The Netherlands; f.x.rosas.plaza@umcg.nl (X.R.P.); j.meijer01@umcg.nl (C.M.); m.vugt@umcg.nl (M.A.T.M.v.V.); s.de.jong@umcg.nl (S.d.J.); 2 Department of Pathology, Erasmus MC Cancer Institute, NL-3015 GD Rotterdam, The Netherlands; a.vanagthoven@erasmusmc.nl; 3 Princess Maxima Center for Pediatric Oncology, NL-3584 CS Utrecht, The Netherlands

**Keywords:** testicular germ cell cancer, microRNA, serum biomarkers, β-HCG, AFP, LDH

## Abstract

Background: LDH (lactate dehydrogenase), AFP (alpha-fetoprotein) and β-HCG (human chorionic gonadotropin) are used in diagnosis and follow-up of testicular germ cell cancer (TGCC) patients. Our aim was to investigate the association between levels of miR-371a-3p, miR-373-3p and miR-367-3p and clinical features in metastatic TGCC. Methods: relative levels of miR-371a-3p, miR-373-3p and miR-367-3p were evaluated in serum of metastatic TGCC patients. A prospectively included and a retrospectively selected cohort were studied (total patient number = 109). Blood samples were drawn at start of chemotherapy and during follow-up. Serum microRNA (miR) levels were determined using the ampTSmiR test. Results: at start of chemotherapy, miR-371a-3p, miR-373-3p and miR-367-3p levels were positively correlated to LDH. The median level of these miRs was higher in patients who developed a relapse after complete biochemical remission (*n* = 34) than in those who had complete durable remission (*n* = 60). Higher levels of miR-367-3p were found in patients with refractory disease (*n* = 15) compared to those who had complete response. miR levels decreased during the first week of chemotherapy in patients with complete response and stayed below threshold after one year of treatment. Conclusion: high miR levels at start of chemotherapy are associated with worse clinical outcome and can assist in early diagnosing of relapses.

## 1. Introduction

Testicular germ cell cancer (TGCC) is rare, but the most common malignancy among young Caucasian men. Even in case of disseminated disease the prognosis is good with cisplatin-based chemotherapy. The tumor markers LDH (lactate dehydrogenase), AFP (alpha-fetoprotein) and β-HCG (human chorionic gonadotropin) produced by some of the cancer cells and measurable in serum contribute to establish the diagnosis, with LDH being the less specific marker. These markers are also used for risk stratification and follow-up of patients with metastatic disease, making them a highly valuable tool for oncologists in daily practice. Nevertheless, overall only ~60% of TGCC patients have elevated markers during the course of their disease [1]. The proportion of patients with elevated tumor markers varies depending on the histological subtype. Around 90% of non-seminoma tumors will be positive for AFP or β-HCG and up to 30% of seminomas will be positive for β-HCG [2]. LDH is elevated in 40–60% of patients regardless of the histological tumor type [3]. Prognosis-based staging of the international germ cell cancer collaborative group (IGCCCG) guide oncologists in deciding treatment for TGCC patients with metastatic disease [2,4]. Notwithstanding the good prognosis of this patient group, ~20% of patients in the intermediate and 30% in poor risk groups, respectively, will die from the disease [5,6]. It is crucial to investigate biomarkers that will help identifying patients who will develop refractory disease or have a higher chance of a tumor relapse after an initial complete remission.

Tumor-derived products can be isolated from blood, with circulating DNA or RNA, exosomes and circulating tumor cells (CTCs) recently being studied in relation to diagnosis and progression of cancer patients [7]. Small non-coding RNAs, also known as microRNAs (miRs), are frequently dysregulated in cancer cells and an oncogenic role of the miR-371 cluster has been described [8]. Specifically, miR-371a-3p and miR-373-3p, both members of the miR-371 cluster, as well as the pluripotency-associated miR-367-3p have been studied in TGCC [9]. These reports have shown the potential utility of the aforementioned miRs as novel biomarkers for diagnosis and follow-up of TGCC patients after initial treatment [10,11,12,13,14,15]. The potential clinical value of the levels of these miRs in blood lie in predicting the presence of viable disease [16], or detecting relapses [17] after chemotherapy for TGCC has been recently described [18,19]. Nonetheless, these studies lack sufficient numbers of poor outcome patients to evaluate the relationship between these miR levels and response to chemotherapy. In addition, the kinetics of these miRs in relation to chemotherapy treatment have not been studied in detail so far.

Therefore, the objectives of this study were to evaluate the potential use of circulating miR-371a-3p, miR-373-3p and miR-367-3p levels to predict relapse or refractory disease in TGCC patients as well as their accuracy at detecting relapses during follow-up after chemotherapy compared to the classical tumor markers. An additional objective was to evaluate the kinetics of the miRs within the first week after start of chemotherapy in patients with metastatic disease was evaluated. Blood samples were collected from metastatic TGCC patients at start of chemotherapy and during treatment and follow-up to assess the levels of miR-371a-3p, miR-373-3p and miR-367-3p using a serum based microRNA detection assay (ampTSmiR test) and correlate these to clinical features.

## 2. Methods

### 2.1. Patients

Two different TGCC patient cohorts with metastatic disease (stages II to IV, according to the Royal Marsden Hospital staging system [20]) were analyzed for this study: a prospectively included cohort was used as a reference group (cohort 1) and a retrospectively selected and constructed cohort of patients with unfavorable outcome (cohort 2). Ethical code number: UMCG2016/041, date of approval: April 12, 2006. All participants gave written informed consent. Patients in cohort 1 received three or four BEP courses (etoposide 100 mg/m^2^, days 1–5, cisplatin 20 mg/m^2^, days 1–5, with/without bleomycin 30 USP, days 2, 8, 15) every three weeks. During the first six days of every course, patients were hydrated with 4L saline per 24h. Serum samples of cohort 1 were taken before start of chemotherapy (week 0) and during chemotherapy treatment at week 1, 3, 6, 8 and after ~3–6 months and ~1y of start of chemotherapy. Cohort 1 was previously described [21]. This study investigated BEP-chemotherapy-induced vascular activation and metabolic changes in metastatic TGCC patients after first-line BEP chemotherapy. The majority of patients in cohort 2 also received three or four BEP courses every three weeks as described above, although several received other platinum based chemotherapy regimens. Patients treated at the UMCG, between February 1998 and October 2013, were included. Patients in cohort 2 were selected from the UMCG local data-biobank of testicular cancer patients upon the occurrence of relapse after first line chemotherapy for metastatic disease or refractory disease during initial chemotherapy. In both cohorts, miR levels were associated with stage, IGCCCG risk group, tumor type, classical tumor markers and clinical outcome.

All serum samples were collected between February 1998 and February 2015, and stored after primary handling at −20 °C. In total, serum samples of 109 patients with a median age of 31 years (range 16–78 years) were included. miR levels of TGCC patients were compared with 104 sera of male healthy donors, as described previously [10]. The normal control serum samples were obtained from Sanquin (Amsterdam, The Netherlands).

### 2.2. Measurement of miR Levels by ampTSmiR Test

The miR purification, quantitative real-time PCR (RT-qPCR) and quality control were as described earlier [10,11]. The miRs were isolated from 50 µL serum using target specific anti-miR magnetic beads as described before [10,17,18]. A MagMaxTM Express-96 robot was used with TaqMan^®^ microRNA ABC Purification Kits (panel A, Thermo Fisher Scientific, Bleiswijk, The Netherlands). The following TaqMan assays were used for cDNA generation and miR level quantification: hsa-miR-371a-3p (catalog ID 002124); hsa-miR-373-3p (catalog ID 000561); hsa-miR-367-3p (catalog ID 000555); ath-miR-159a (catalog ID 000338) and hsa-miR-30b-5p (catalog ID 000602) (Thermo Fisher Scientific). A TaqMan MicroRNA RT KIT (Thermo Fisher Scientific) was used to reverse transcribe specifically targeted purified miR (5 µL in elution buffer) into miR specific cDNA. Subsequently, a 13 cycle pre-amplification step was performed using 2× TaqMan Preamp Master mix (4488593, detailed protocol by supplier Thermo Fisher Scientific) and 20× TaqMan MicroRNA Assays. Thermal-cycling conditions: 95 °C for 10 min., next 13 cycles of 95 °C for 15 s and 60 °C for 1 min. miRNA levels were determined on a TaqMan 7500 Real-Time PCR system in 1.5 µL of cDNA (Thermo Fisher Scientific).

### 2.3. Quality Control

A non-human miR spike-in ath-miR-159a was added for quality control of miR isolation and cDNA generation and was used to correct for input. All sera were visually inspected and miR-451 was used to control for hemolysis, no samples were discarded from analysis. No samples had to be excluded due to poor miR recovery. For normalization the mean levels of the endogenous reference miR (miR-30b-5p) was used as described before [10,11,16]. In each cDNA synthesis experiment, a dilution series of purified miR of the TCam-2 seminoma cell line was included for quality control and qPCR efficiency and interplate calibration. As negative control (no template control), elution buffer was added instead of purified miR.

### 2.4. Evaluation

The target miR level per sample was determined according to the delta Ct method. Relative miR levels are depicted as 2^˄^(−delta Ct (miR of interest minus reference miR) × 10^4^. A cutoff value of ≥2.0 time of the highest level observed in the healthy donor group was used to set a threshold as described earlier [11]. This threshold was used to select patients for the kinetics analysis.

### 2.5. Statistical Analysis

Data are presented as median and range. For comparison between groups the Mann-Whitney U test was applied. To study the correlation between tumor markers and miR relative levels, Spearman correlation coefficient was used. To test significant differences in miR levels after start of chemotherapy, repeated measures ANOVA was used. Two-sided *p*-values < 0.05 were considered significant. Statistical analyses were performed in SPSS Statistics 22.0 (IBM SPSS Inc, Armonk, NY USA) and GraphPad Prism 7.0 (GraphPad Inc, San Diego, CA, USA).

## 3. Results

### 3.1. Patients

**Figure 1:**
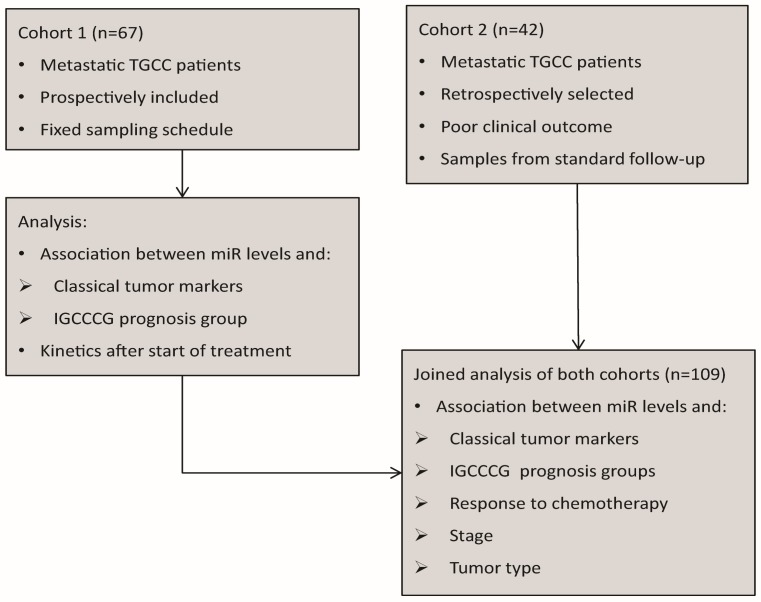
Schematic view of cohort 1, as described before (see Methods) and cohort 2 and the analyses performed.

This study used two different cohorts, and analysis was performed in two steps (Figure 1). Cohort 1 (prospectively included) was used as a reference group since it stands as a representative sample of metastatic TGCC with the advantage of having a fixed sampling schedule. Because of the good prognosis of TGCC, cohort 1 lacked enough relapse and refractory disease cases. Therefore, cohort 2 (retrospectively selected) was added to this analysis to enrich for cases with unfavorable outcome. Cohort 2 did not have a fixed sampling schedule for each patient besides the standard follow-up for metastatic TGCC.

Cohort 1 consisted of 67 patients who were prospectively included. Eligibility criteria were diagnosis of disseminated TGCC and BEP chemotherapy as first line treatment. Characteristics are described in Table 1. The majority was stratified in the good risk group (83.6%) according to the IGCCCG classification [4], and only 14.9% and 1.5% belonged to the intermediate and poor risk groups, respectively. In total, six patients had a relapse and one had refractory disease.

Forty-two patients were retrospectively included in cohort 2. Eligibility criteria were diagnosis of disseminated TGCC and presence of relapse after completion of chemotherapy or refractory disease (defined as an increase in marker level during or at completion of first line chemotherapy). Characteristics are shown in Table 1. At start of chemotherapy, 11 patients were classified in the good (26.2%), 15 in the intermediate (35.7%) and 16 in the poor IGCCCG risk group (38.1%). Twenty-eight patients (66.7%) had a relapse after an initial good biochemical response on chemotherapy and 14 patients had refractory disease on chemotherapy (33.3%).

### 3.2. Association between miR Levels, Tumor Markers and IGCCCG Risk Groups in Cohort 1

Serum levels of LDH, AFP and β-HCG were compared with levels of miR-371a-3p, miR-373-3p and miR-367-3p before start of chemotherapy. LDH levels had a positive correlation with levels of all three miRs (miR-371a-3p, *p* ≤ 0.001; miR-373-3p, *p* ≤ 0.001; miR-367-3p, *p* = 0.005) (Supplementary Table S1). β-HCG positively correlated with miR-371a-3p only (*p* = 0.045), whereas no correlation was found between AFP and any of the analyzed miR levels (Supplementary Table S1).

Analysis of the miR levels before start of chemotherapy and IGCCCG risk stratification showed that median miR-371a-3p levels were higher in patients from intermediate and poor risk groups compared to patients belonging to the good risk group (*p* = 0.041) (Supplementary Figure S1A–C, Supplementary Table S2).

### 3.3. Association between miR Levels, Classical Tumor Markers and Clinical Features in Cohort 1 and 2 Combined

**Figure 2:**
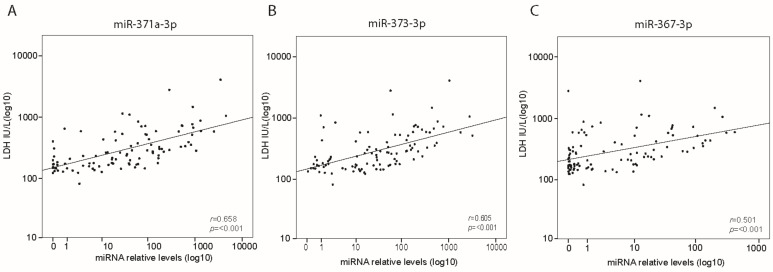
Spearman correlation of relative levels of miR-371a-3p (**A**), miR-373-3p (**B**) and miR-367-3p (**C**) and LHD before start of chemotherapy in cohort 1 and 2 combined (*n* = 101).

**Figure 3:**
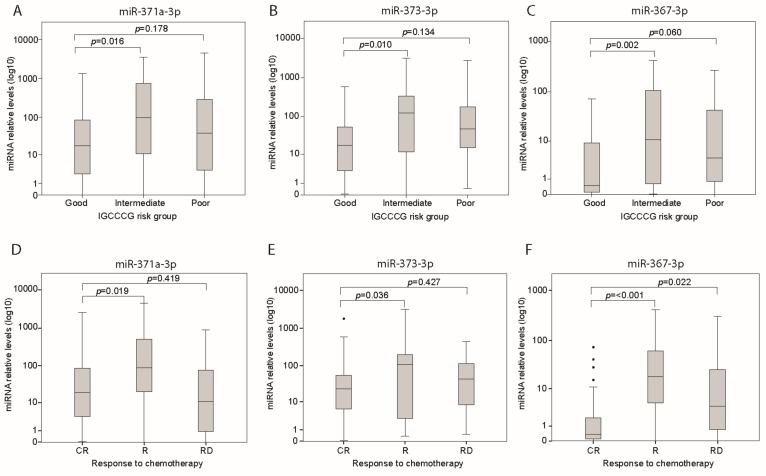
Relative levels of miR-371a-3p (**A**), miR-373-3p (**B**) and miR-367-3p (**C**) before start of chemotherapy in cohort 1 and 2 combined in good (*n* = 67), intermediate (*n* = 25) or poor risk (*n* = 17) IGCCCG group. Relative levels of miR-371a-3p (**D**), miR-373-3p (**E**) and miR-367-3p (**F**) before start of chemotherapy in cohort 1 and 2 with complete response (CR, *n* = 60), relapse (R, *n* = 34) or refractory disease (RD, *n* = 15). The parameter p denotes Mann Whitney U test significance.

Analysis of both cohorts combined (Figure 1) showed a positive correlation between LDH and miR-371a-3p, miR-373-3p and miR-367-3p (all *p* ≤ 0.001) (Figure 2A–C, Supplementary Table S3). β-HCG was positively correlated with miR-371a-3p (*r* = 0.295; *p* = 0.003) and miR-373-3p (*r* = 0.263; *p* = 0.007) but not miR-367-3p (*r* = 0.143; *p* = 0.151) levels (Supplementary Table S3). AFP was positively correlated with miR-367-3p only (*r* = 0.194; *p* = 0.049) (Supplementary Table S3). The miR levels were evaluated comparing tumor histology: median levels of miR-371a-3p (*p* = 0.037) and miR-373-3p (*p* = 0.005) were higher in seminoma than in non-seminoma patients (Supplementary Figure S2A,B, Supplementary Table S4), while miR-367-3p levels did not differ between both tumor types (Supplementary Figure S2C, Supplementary Table S4). Analysis of cohort 1 and 2 combined confirmed significantly higher median levels of miR-371a-3p (*p* = 0.016), miR-373-3p (*p* = 0.010) and miR-367-3p (*p* = 0.002) in the intermediate risk group compared with the good risk group (Supplementary Table S5, Figure 3A–C). Median levels of miR-371a-3p, miR-373-3p and miR-367-3p were not higher in patients from the poor risk group compared to the good risk group (Supplementary Table S5, Figure 3A–C). Nonetheless, analysis including non-seminoma patients only showed higher median miR levels of miR-373-3p (*p* = 0.030) and miR-367-3p (*p* = 0.012) in poor prognosis compared to good prognosis patients (Supplementary Table S6). Analysis of miR levels comparing different stages of disease showed that patients diagnosed with stage III and IV had higher miR-367-3p levels (*p* = 0.007 and *p* = 0.004, respectively) than those who had stage II disease (Supplementary Figure S2F, Supplementary Table S7). Levels of miR-371a-3p and miR-373-3p did not statistically differ across the stages of disease (Supplementary Figure S2D,E, Supplementary Table S7). From the 89 patients with evaluable LDH, AFP and β-HCG, nine (10.1%) were negative for all three classical tumor markers. Interestingly, three of these nine patients were positive for at least one of the miRs.

In patients from cohort 1 and 2 who underwent a postchemotherapy RPLND miR-371a-3p were not elevated at time of the operation in the patients with fibrosis/necrosis or teratoma in their resected lesions. In three of four patients who underwent a postchemotherapy RPLND, and found to have vital tumor in their resected lesion, the miR 371a-3p was elevated.

### 3.4. Use of miR Levels to Predict Relapse after a Complete Response, or Refractory Disease

The value of the miR levels before start of chemotherapy to predict relapse or refractory disease was also evaluated. Analysis was performed using cohorts 1 and 2 combined (Figure 1) to enrich for cases of relapse and refractory disease. Before start of chemotherapy, the median levels of miR-371a-3p (*p* = 0.019), miR-373-3p (*p* = 0.036) and miR-367-3p (*p* ≤ 0.001) in patients with a durable complete remission after chemotherapy were lower than the levels in patients who developed a relapse after complete remission (Figure 3D–F, Supplementary Table S8). The median miR-367-3p level in patients with refractory disease was higher than the level in patients who had a complete response (*p* = 0.022). We also evaluated how many patients had elevated miR levels at time of diagnosis of relapse, or refractory disease. From the total of 34 patients with relapse after complete remission, 21 had available samples at moment of relapse. Twelve patients had at least one elevated miR when the relapse was diagnosed. When using miR-371a-3p patients nine patients did not have elevated miR-371a-3p levels at time of their relapse resulting in a sensitivity of 57%. Specificity could not be calculated based on the defined selection of the patients within this cohort (absence of cases with elevated miR-371a-3p without presence of a relapse). Details of these nine patients with a relapse after a biochemical complete remission without an elevated miR-371a-3p level at time of the relapse are listed in Supplementary Table S9. In seven patents elevated tumor markers (AFP in three, HCG in two, LDH in two) were identified at the moment of relapse. In two patients, none of the biomarkers were elevated and both had a retroperitoneal relapse. As indicated in Supplementary Table S9, two patients had only teratoma at the relapsed site, explaining their negative miR-371a-3p level. In the two patients with seminoma (and elevated LDH at time of relapse) no histology of the relapsed site was available. In five patients histological examination of the relapsed site did contain other malignant components than teratoma [embryonal carcinoma and yolk sac tumor].

Because the false positive elevated miR-371a-3p could not be estimated, the specificity was not calculated. Eight of the patients with a relapse presented their relapse with elevated miRs earlier than classical tumor markers, and in two other patients miRs were elevated, while the classical tumor markers remained negative. From thirteen out of fifteen patients with refractory disease, samples during follow-up were available. Twelve of these patients (86.6%) had at least one elevated miR during the follow-up period and in six patients elevated miR levels were observed, while classical tumor markers were decreasing.

### 3.5. Kinetics of miR Levels during Chemotherapy in Cohort 1

**Figure 4:**
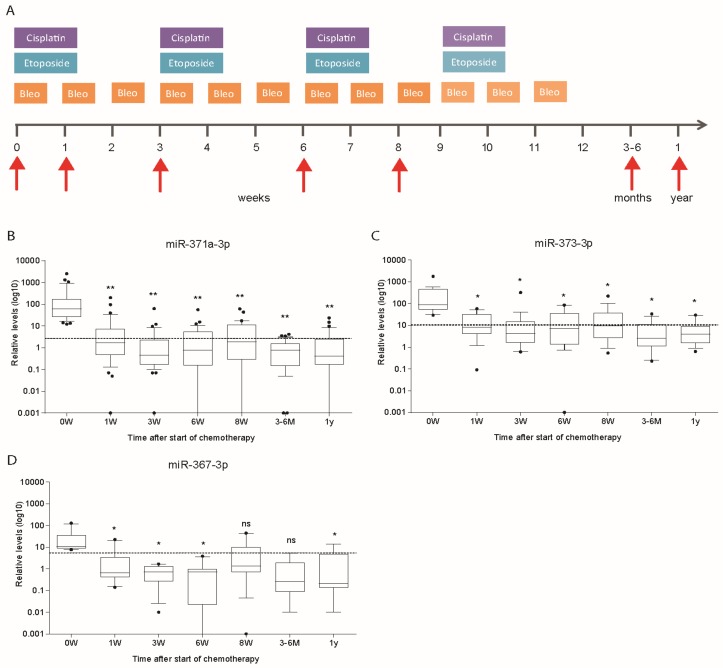
Bleomycin, etoposide and cisplatin (BEP) containing chemotherapy schedule (3 or 4 courses) as well as sampling time points (red arrows) of cohort 1 (**A**). Follow-up of relative level of miR-371a-3p (*n* = 39) (**B**), miR-373-3p (*n* = 19) (**C**) and miR-367-3p (*n* = 10) (**D**) after start of chemotherapy. Patients with relative miR levels above the described threshold at start of treatment are depicted. Repeated measures ANOVA was used to test significance (* *p* < 0.05, ** *p* < 0.005).

Kinetics of miR levels were evaluated in patients from cohort 1 who had a miR level above the previously described threshold [11]. Thirty-nine (58.2%) patients had levels above the threshold of miR-371a-3p, nineteen (28.3%) of miR-373-3p and ten (14.9%) of miR-367-3p. Follow-up of miR levels after initiation and during treatment showed a rapid decrease of miR-371a-3p, miR-373-3p and miR-367-3p levels (Figure 4A–D). The sharp decrease after the initiation of the first cycle of BEP administration was seen for all three miRs (Figure 4B–D). The miR levels fell below threshold in most of the patients during the chemotherapy treatment.

Analysis of the kinetics of miR levels during the first week upon treatment was used to estimate half-life of miR-371-3p. Data were available from twelve patients belonging to cohort 1. This subset of patients was selected upon positivity at start of chemotherapy, measurable miR levels at 24 and eventually 48 h, and absence of later peaks of miR-371a-3p. The estimated median half-life of miR-371-3p was 27 h (range 6–86 h).

### 3.6. The MiR Levels during Follow-up of Patients

**Figure 5:**
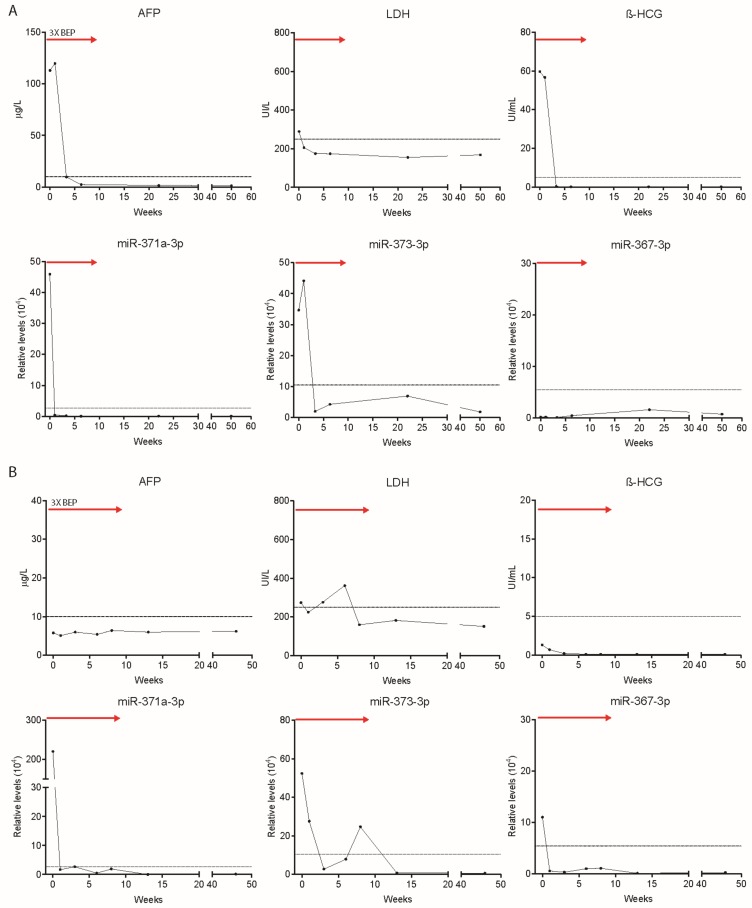
Levels of serum markers alpha-fetoprotein (AFP) (μg/L), LDH (UI/L) β-HCG (UI/mL) and relative levels of miR-371a-3p, miR-373-3p and miR-367-3p at start of chemotherapy, during treatment (red arrow) and follow-up of TGCC case 1 (**A**) and case 2 (**B**). Red arrows indicate chemotherapy duration (three courses of BEP).

Illustrative and representative cases of TGCC patients with metastatic disease who presented with high levels of at least one of the miRs were studied individually after the start of chemotherapy and during follow-up. Classical tumor markers, as well as miR levels from two patients (cases 1 and 2 from cohort 1) who had a complete response at last follow-up after BEP chemotherapy are outlined in Figure 5. Patient depicted as case 1 (non-seminoma (embryonal carcinoma and teratoma), stage II, intermediate IGCCCG risk group) had high levels of AFP and β-HCG at start of chemotherapy, after which they normalized. Levels of miR-371a-3p and miR-373-3p followed the same pattern as AFP and β-HCG, with high levels at the start of treatment and normalization after chemotherapy (Figure 5A). Case 2 represents a patient diagnosed with stage 2 non-seminoma (embryonal carcinoma and seminoma) who was negative for the classical tumor markers. Nevertheless, miR-371a-3p, miR-373-3p and miR-367-3p levels were above the upper limit of normal and rapidly declined after first BEP administration (Figure 5B). Supplementary Figure S3A shows levels of AFP and LDH of case 3 (cohort 2): a patient who was diagnosed with mixed TGCC. At the moment of diagnosis, the patient had stage 2 disease and was classified as intermediate risk according to the IGCCCG. The patient underwent four cycles of BEP chemotherapy, after which AFP levels never normalized, and was eventually diagnosed with refractory disease. Interestingly, levels of miR-371a-3p, miR-373-3p and miR-367-3p dramatically increased at week 8, while AFP was slowly decreasing, pointing at the early detection of this case of refractory disease only with the use of miRNA levels. Supplementary Figure S3B represents case 4 (cohort 2); a seminoma patient (stage 2, good risk) with elevated LDH only at moment of diagnosis who was treated with four courses of a carboplatin-based chemotherapy regimen. Only miR-371a-3p and miR-367-3p were elevated at the start of treatment, and although levels fluctuated, at week 26 all three miR levels increased drastically. A relapse was detected based on CT scan at week 39, when the three miRs showed very high levels in contrast to the classical serum bio-markers. Four courses of BEP chemotherapy were administered to the patient. During this second line of chemotherapy, miR levels decreased but fluctuated around the normal threshold. The disease of this patient ultimately progressed with a rise in all tumor markers and miR-371a-3p, miR-373-3p and miR-367-3p levels followed this increase. Ultimately, the patient died of the disease.

## 4. Discussion

This study indicates that serum levels of members of the miR-371 cluster and miR-367-3p before start of chemotherapy correlate with various clinical features in metastatic TGCC patients, including disease burden. TGCC patients belonging to the good IGCCCG prognosis group showed lower median miR levels compared to the intermediate and poor risk groups. Patients who developed a relapse after complete remission had higher levels of miR-371a-3p, miR-373-3p and miR-367-3p before start of chemotherapy than patients who had a durable complete remission. Furthermore, median level of miR-367-3p at the start was higher in metastatic patients who developed refractory disease. Levels of miR-371a-3p, miR-373-3p and miR-367-3p decreased below threshold shortly after start of chemotherapy and after doing so those levels remained below threshold at one year after treatment completion in the favorable responding patients.

Recent studies have confirmed the diagnostic value of miR-371a-3p, miR-373-3p and miR-367-3p levels in germ cell tumors [10,11]. Patients with a TGCC had significantly higher miR-371a-3p, miR-373-3p and miR-367-3p levels than healthy donors without cancer or patients with other malignancies. Furthermore, two recent studies found that pre-chemotherapy miR levels were associated with tumor stage [12,16]. Our data indicated that TGCC patients with stage III and IV had a higher median level of miR-367-3p than stage II, but no differences in miR-371a-3p and miR-373-3p levels. A limitation of our study is the absence of samples before orchiectomy, since the reduction in tumor burden after surgery could influence miR levels. In the recent reported study of Dieckmann et al. it was shown that detection of miR-371a-3p discriminates patients with localized disease from those with metastases at the time of diagnosis with a sensitivity and specificity of 83.4% and 60.1%, respectively [12]. The sensitivity of that study was higher than reported by us here, being at the time of start of chemotherapy. It is known that orchiectomy results in a decrease in miR levels in many patients. Of interest is that among the few clinical stage II patients [21.3%] in the Dieckmann et al. study the miR level dropped to normal postoperatively (i.e., possible false negative finding). In our study, a much higher percentage of patients (*n* = 66; 60.6%) had clinical stage II disease. Of these 35 patients (53.0%) had a positive miR-371a-3p levels at start of chemotherapy. This might explain the lower percentage (58.2%) of positive miR-371a-3p levels of the whole group at time of the start of chemotherapy. This is in line with a previous study showing elevated miR-371a-3p levels in 51.7% of the cases [19]. It is a tempting hypothesis that these miR-371a-3p patients are clinically over-staged. This is in line with surgical studies documenting staging error in approximately 20% of clinical stage II patients [22].

Analysis of miR levels in relation with classical tumor markers showed that LDH positively correlated with miR-371a-3p, miR-373-3p and miR-367-3p levels. LDH is regarded as an indirect measure of tumor burden in TGCC patients [23]. This suggests that miR level can serve as an indirect measure of tumor burden as well. In line with our findings, a recent report demonstrated that the combined use of miR-371a-3p and miR-373-3p levels can identify the presence of vital tumor after retroperitoneal lymph node dissection (RPLND) with a 100% sensitivity and 58% specificity [16]. In the current reported series, testicular cancer patients with only teratoma in their postchemotherapy RPLND [*n* = 28] we did not find elevated miR 371a-3p levels at time resection of their residual disease in line with previous studies [16].

Median miR levels before start of chemotherapy were higher in patients from the intermediate and poor prognosis groups compared to good risk IGCCCG group. Nevertheless, it is not possible to identify patients who are at higher risk of developing relapse or refractory disease since poor risk group patients still have a 50% five-year overall survival. This means that there is a considerable proportion of patients within this subset that will respond well to chemotherapy and have a favorable outcome [6]. Before start of chemotherapy, patients who developed a relapse after a complete biochemical response had higher miR-371a-3p, miR-373-3p and miR-367-3p median levels than patients without relapse. miR-367-3p was also higher in those who developed refractory disease. These data illustrate that miR evaluation at start of chemotherapy will help to identify patients who are at higher risk of developing relapse or refractory disease. Interestingly, 10% of relapse cases were detected with miR levels in patients with negative classical tumor markers. In addition, in 40% of patients who developed refractory disease miR levels were increasing while classical tumor markers were still decreasing. The advantage of suspecting an unfavorable outcome before or earlier during treatment is to give oncologists the possibility to adjust or intensify the chemotherapy treatment earlier in the course of the disease in an effort to improve outcome. At time of a relapse after complete remission, 21 patients could be evaluated. When using miR-371a-3p patients, nine patients did not have elevated miR-371a-3p levels at time of their relapse resulting in a sensitivity of 57%. Two of these relapsed patients had only teratoma at their relapsed site. These two patients had a negative miR level, as expected since teratoma does not result in elevated serum miR-371a-3p level. Specificity could not be meaning-full estimated based on the low numbers and the defined selection of the patients within this particular cohort (i.e., absence of cases with elevated miR-371a-3p without presence of a relapse).

The levels of miR-371a-3p, miR-373-3p and miR-367-3p upon start of chemotherapy revealed a rapid decrease during the first week of treatment. Furthermore, these levels remained below threshold up to 1 year after chemotherapy completion. In line with our data, Dieckman and co-workers showed that miR-371-3p levels were lower after the first cycle of chemotherapy compared to pre-chemotherapy levels in patients with metastasis [12]. However, to our knowledge our study is the first that analyzed in such detail the dynamics of miR-371a-3p, miR-373-3p and miR-367-3p levels during and after treatment in a cohort of metastatic TGCC. Moreover, we gained insight in serum miR-371a-3p half-life after start of BEP chemotherapy, which varied largely between patients and was estimated to be approximately 27 h.

In conclusion, we show miR-371a-3p, miR-373-3p and miR-367-3p to be potentially valuable biomarkers in patients with metastatic TGCC. These miRs should be further evaluated before being incorporated as part of clinical practice to identify patients with a higher risk of developing a relapse after a complete biochemical response or who develop refractory disease. These miRs may have an impact on the initial treatment of newly diagnosed TGCC patients with metastatic disease. The use of these miRs could potentially identify TGCC patients with a worse prognosis. In addition, our results demonstrate that miRs could be of value to follow-up TGCC patients who are negative for the classical tumor markers.

## Figures and Tables

**Table 1:** Clinical characteristics of cohorts 1 and 2.

Variable	Number (%)	
All Patients	Cohort 1 67 (100%)	Cohort 2 42 (100%)
**Tumor type (histology)**		
-Non seminoma	54 (80.3)	31 (73.8)
Embryonal carcinoma ^$^	44	19
Choriocarcinoma	6	9
Yolk sac	23	17
Teratoma	26	25
Seminoma	30	17
-Pure Seminoma	13 (19.7)	10 (23.8)
-Extragonadal		1 (2.4)
**IGCCCG Risk Group**		
-Good	56 (83.6)	11 (26.2)
-Intermediate	10 (14.9)	15 (35.7)
-Poor	1 (1.5)	16 (38.1)
**Response to Chemotherapy**		
-Durable complete response (NED)	60 (89.5)	-
-Relapse after complete response (NED)	6 (9)	28 (66.7)
-Refractory disease	1 (1.5)	14 (33.3)
**Stage (Metastatic Disease)**		
-II	54 (80.6)	12 (28.6)
-III	6 (9)	6 (14.3)
-IV	7 (10.4)	24 (57.1)
**RPLND [Post Chemotherapy]**		
-Yes	32 (47.8)	16 (38.1)
Necrosis/fibrosis	11	5
Teratoma	20	8
Vital carcinoma	1	3
-No	35 (52.2)	26 (61.8)
**β-HCG Measurements**	61 (91)	41 (98)
-Positive	33 (54)	31 (76)
-Negative	28 (46)	10 (24)
**AFP Measurements**	62 (93)	42 (100)
-Positive	34 (55)	27 (64)
-Negative	28 (45)	15 (36)
**LDH Measurements**	60 (90)	41 (98)
-Positive	19 (32)	32 (78)
-Negative	41 (68)	9 (22)

IGCCCG: international germ cell cancer collaborative group; RPLND: retroperitoneal lymph node dissection. β-HCG (human chorionic gonadotropin), AFP (alpha-fetoprotein) and LDH (lactate dehydrogenase). ^$^ Different histological components of non-seminoma in removed affected testicle. NED (No evidence of disease).

## Supplementary Materials

The following are available online at https://www.mdpi.com/2073-4409/8/10/1221/s1. Table S1: Correlation of LDH, AFP and β-HCG levels with miR-371a-3p, miR-373-3p and miR-367-3p relative levels before start of chemotherapy in cohort 1. Table S2: miR-371a-3p, miR-373-3p and miR-367-3p relative levels before start of chemotherapy in cohort 1 with good versus intermediate/poor prognosis. Table S3: Correlation of LDH, β-HCG and AFP levels with miR-371a-3p, miR-373-3p and miR-367-3p relative levels before start of chemotherapy in both cohorts 1 and 2 combined. Table S4: miR-371a-3p, miR-373-3p and miR-367-3p relative levels before start of chemotherapy in cohort 1 and 2 combined in non-seminoma and seminoma (*n* = 108 baseline measurements). Table S5: miR-371a-3p, miR-373-3p and miR-367-3p relative levels before start of chemotherapy in cohort 1 and 2 combined in good, intermediate and poor risk IGCCCG groups (*n* = 109 baseline measurements). Table S6: miR-371a-3p, miR-373-3p and miR-367-3p relative levels before start of chemotherapy in non-seminoma patients from cohort 1 and 2 combined in good, intermediate and poor risk IGCCCG groups (*n* = 85 baseline measurements). Table S7: miR-371a-3p, miR-373-3p and miR-367-3p relative levels before start of chemotherapy in cohort 1 and 2 combined in stage II, III and IV (*n* = 109 baseline measurements). Table S8: miR-371a-3p, miR-373-3p and miR-367-3p relative levels before start of chemotherapy comparing patients of cohort 1 and 2 combined with complete response, relapse and refractory disease (*n* = 109 baseline measurements). Table S9: Details of the 9 patients with a relapse after a biochemical complete remission without an elevated miR-371a-3p level at time of the relapse. Figure S1: Relative levels of miR-371a-3p (**A**), miR-373-3p (**B**) and miR-367-3p (**C**) before start of chemotherapy in cohort 1 comparing good (*n* = 56) and intermediate/poor risk (*n* = 11) IGCCCG groups. p denotes Mann Whitney U test significance. Figure S2: Relative level of miR-371a-3p (**A**), miR-373-3p (**B**) and miR-367-3p (**C**) before start of chemotherapy in cohort 1 and 2 combined comparing non-seminoma (*n* = 85) and seminoma (*n* = 23) tumors. Relative level of miR-371a-3p (**D**), miR-373-3p (**E**) and miR-367-3p (**F**) before start of chemotherapy in cohort 1 and 2 combined comparing stage II (*n* = 66), stage III (*n* = 12) and stage IV (*n* = 31) tumors. p denotes Mann Whitney U test significance. Figure S3: Levels of serum markers AFP (μg/L), LDH (UI/L) β-HCG (UI/mL) and relative levels of miR-371a-3p, miR-373-3p and miR-367-3p at start of chemotherapy, during treatment (red and blue arrows) and follow-up of TGCC case 3 (**A**) and case 4 (**B**). Red and blue arrows indicate chemotherapy duration 4 × BEP (bleomycin, etoposide, and cisplatin), 4 × TIP (paclitaxel, ifosfamide and cisplatin), 4 × COC (cyclophosphamide, vincristine and carboplatin).
