# Supplementary material for: miR-371a-3p, miR-373-3p and miR-367-3p as Serum Biomarkers in Metastatic Testicular Germ Cell Cancers Before, During and After Chemotherapy
Source: Cells. 2019 Oct 8;8(10):1221. doi: 10.3390/cells8101221 (PMC6830325; doi:10.3390/cells8101221)
Supplement: Supplementary file 1 [file cells-08-01221-s001.pdf]

Supplementary tables on miR levels, classical tumor markers and correlations

Supplementary table 1. Correlation of LDH, AFP and ß-HCG levels with miR-371a-3p, miR-373-3p and miR-367-3p relative levels before start of chemotherapy in cohort 1.

Tumor marker	miR-371a-3p	miR-373-3p	miR-367-3p
LDH (*n*=60)			
- Correlation coefficient	0.694	0.587	0.359
- *p**	<0.001	<0.001	0.005
ß-HCG (*n*=61)			
- Correlation coefficient	0.258	0.076	0.079
- *p**	0.045	0.558	0.543
AFP (*n*=62)			
- Correlation coefficient	-0.005	-0.063	0.111
- *p**	0.968	0.628	0.391

LDH: lactate dehydrogenase; AFP: alpha-fetoprotein; ß-HCG: human chorionic gonadotropin.
*Spearman correlation coefficient significance, 95% confidence interval

Supplementary table 2. miR-371a-3p, miR-373-3p and miR-367-3p relative levels before start of chemotherapy in cohort 1 with good versus intermediate/poor prognosis.

	Good prognosis *n*=56	Intermediate/Poor prognosis *n*=11	*p**
miR-371a-3p			
- Median	15.3	73.2	
- Range	0-1312.7	0.09-2538.3	0.041
miR-373-3p			
- Median	16.7	28.5	
- Range	0.06-580.8	0.6-1778.3	0.417
miR-367-3p			
- Median	0.3	0.4	
- Range	0-73.5	0-128.4	0.091

Prognostic-based groups according to the IGCCCG classification
*Mann Whitney U test significance

Supplementary table 3. Correlation of LDH, ß-HCG and AFP levels with miR-371a-3p, miR-373-3p and miR-367-3p relative levels before start of chemotherapy in both cohorts 1 and 2 combined.

**Tumor marker**	**miR-371a-3p**	**miR-373-3p**	**miR-367-3p**
LDH (*n*=101)			
- Correlation coefficient	0.658	0.605	0.501
- *p**	<0.001	<0.001	<0.001
ß-HCG (*n*=102)			
- Correlation coefficient	0.295	0.263	0.143
- *p**	0.003	0.007	0.151
AFP (*n*=104)			
- Correlation coefficient	0.051	0.016	0.194
- *p**	0.605	0.868	0.049
LDH: lactate dehydrogenase; AFP: alpha-fetoprotein; ß-HCG: human chorionic gonadotropin. *Spearman correlation coefficient significance, 95% confidence interval			

Supplementary table 4. miR-371a-3p, miR-373-3p and miR-367-3p relative levels before start of chemotherapy in cohort 1 and 2 combined in non-seminoma and seminoma (*n*=108 baseline measurements).

	**Non-seminoma *n*=85**	**Seminoma *n*=23**	***p****
miR-371a-3p			
- Median	20.4	94.8	
- Range	0-4535.7	0.1-3481.9	0.037
miR-373-3p			
- Median	19.9	124.7	
- Range	0.06-3174.1	0.8-1031.9	0.005
miR-367-3p			
- Median	1.0	4.8	
- Range	0-416.0	0-93.0	0.474
*Mann Whitney U test significance			

Supplementary table 5. miR-371a-3p, miR-373-3p and miR-367-3p relative levels before start of chemotherapy in cohort 1 and 2 combined in good, intermediate and poor risk IGCCCG groups (*n*=109 baseline measurements).

	**Good prognosis *n*=67**	**Intermediate prognosis *n*=25**	**Poor prognosis *n*=17**	**Intermediate vs good**	**Poor vs good**
miR-371a-3p					
- Median	17.8	97.6	40.5		
- Range	0-1312.7	0-3481.9	0-4535.7		
- *p**				0.016	0.178
miR-373-3p					
- Median	20.5	127.0	48.3		
- Range	0.06-580.8	0.6-3174.1	0.5-2779.5		
- *p**				0.010	0.134
miR-367-3p					
- Median	0.5	10.9	4.0		
- Range	0-73.5	0.02-416	0-265.5		
- *p**				0.002	0.060

Prognostic-based groups according to the IGCCCG classification
*Mann Whitney U test significance

Supplementary table 6. miR-371a-3p, miR-373-3p and miR-367-3p relative levels before start of chemotherapy in non-seminoma patients from cohort 1 and 2 combined in good, intermediate and poor risk IGCCCG groups (*n*=85 baseline measurements).

	**Good prognosis *n*=50**	**Intermediate prognosis *n*=19**	**Poor prognosis *n*=16**	**Intermediate vs good**	**Poor vs good**
miR-371a-3p					
- Median	13.7	106.6	38.2		
- Range	0-1063.6	0-2538.3	0-4535.7		
- *p**				0.005	0.089
miR-373-3p					
- Median	12	127	38.4		
- Range	0.06-224	0.6-3174.1	0.5-2779.5		
- *p**				0.003	0.030
miR-367-3p					
- Median	0.3	34.8	4.2		
- Range	0-73.5	0.02-416	0-265.5		
- *p**				0.003	0.012

Prognostic-based groups according to the IGCCCG classification
*Mann Whitney U test significance

Supplementary table 7. miR-371a-3p, miR-373-3p and miR-367-3p relative levels before start of chemotherapy in cohort 1 and 2 combined in stage II, III and IV (*n*=109 baseline measurements).

	**Stage II *n*=66**	**Stage III *n*=12**	**Stage IV *n*=31**	**Stage III vs stage II**	**Stage IV vs stage II**
miR-371a-3p					
- Median	22.9	76.9	40.5		
- Range	0-2538.3	0.1-1337.1	0-4535.7		
- *p**				0.091	0.230
miR-373-3p					
- Median	25.6	110.6	29.5		
- Range	0.06-3174.1	0.8-482.8	0.5-2779.5		
- *p**				0.455	0.255
miR-367-3p					
- Median	0.99	8.4	7.6		
- Range	0-416.0	0.06-416	0-265.5		
- *p**				0.007	0.004
*Mann Whitney U test significance					

Supplementary table 8. miR-371a-3p, miR-373-3p and miR-367-3p relative levels before start of chemotherapy comparing patients of cohort 1 and 2 combined with complete response, relapse and refractory disease (*n*=109 baseline measurements).

	**Complete response** **_n_=60**	**Relapse** **_n_=34**	**Refractory disease** **_n_=15**	**Relapse vs complete response**	**Refractory disease vs complete response**	**Relapse vs refractory disease**
miR-371a-3p						
- Median	19.1	89	10.7			
- Range	0-2538.27	0-4535.7	0-901.1			
- *p**				0.019	0.419	0.039
miR-373-3p						
- Median	23.7	109.5	43.2			
- Range	0.06-1778.3	0-3173.8	0.5-442.6			
- *p**				0.036	0.427	0.429
miR-367-3p						
- Median	0.3	18.1	3.9			
- Range	0-73.5	0-416	0-305.3			
- *p**				<0.001	0.022	0.298

*Mann Whitney U test significance

Supplementary table 9: Details of the nine patients with a relapse after a biochemical complete remission without an elevated miR-371a-3p level at time of the relapse.

Patient	Histology primary tumor	Site of relapse	Elevated tumor marker at time of relapse	Only teratoma present at relapsed site
1	NS [EC,YS]	Retroperitoneum	AFP	No
2	NS [EC, T]	Retroperitoneum	none	Yes
3	NS [EC,C]	Mediastinum	HCG	No
4	NS [EC,YS,T]	Liver	AFP	No
5	S	Retroperitoneum	LDH	NA
6	NS [EC,T]	Lungs	HCG	No
7	S	Mediastinum	LDH	NA
8	NS [EC,T]	Retroperitoneum	none	Yes
9	NS [YS,S,T]	Retroperitoneum	AFP	No

Embryonal carcinoma [E], Choriocarcinoma [C], Yolk sac [YS], Teratoma [T], Seminoma [S]. Not applicable [NA]

Supplementary Figure 1

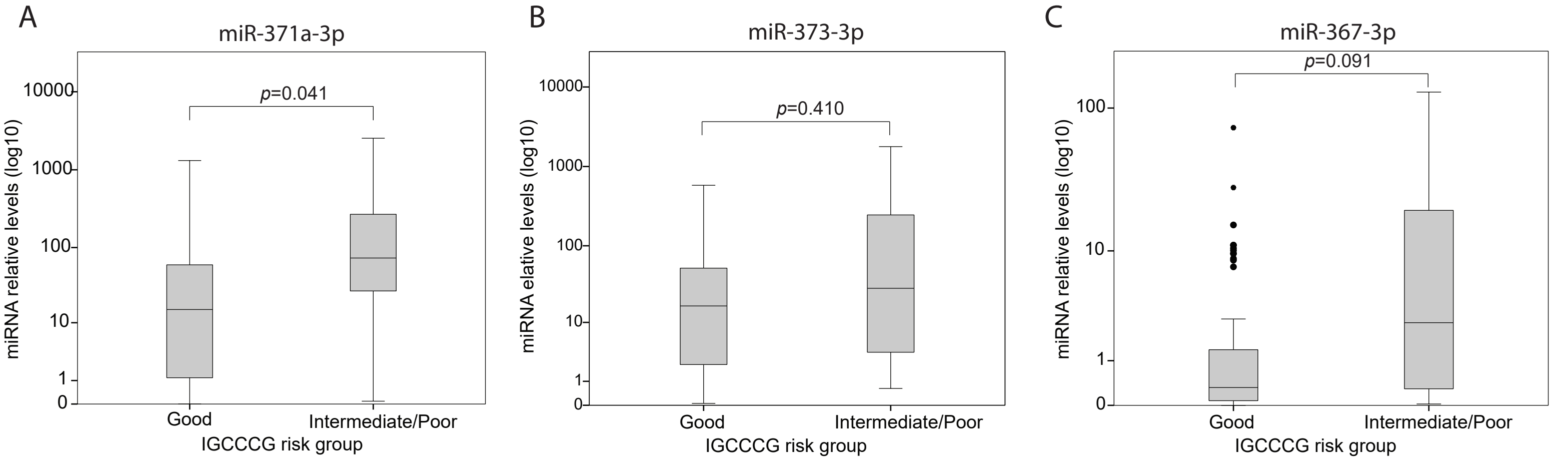

Relative levels of miR-371a-3p (A), miR-373-3p (B) and miR-367-3p (C) before start of chemotherapy in cohort 1 comparing good (n=56) and intermediate/poor risk (n=11) IGCCCG groups. p denotes Mann Whitney U test significance.

Supplementary Figure 2

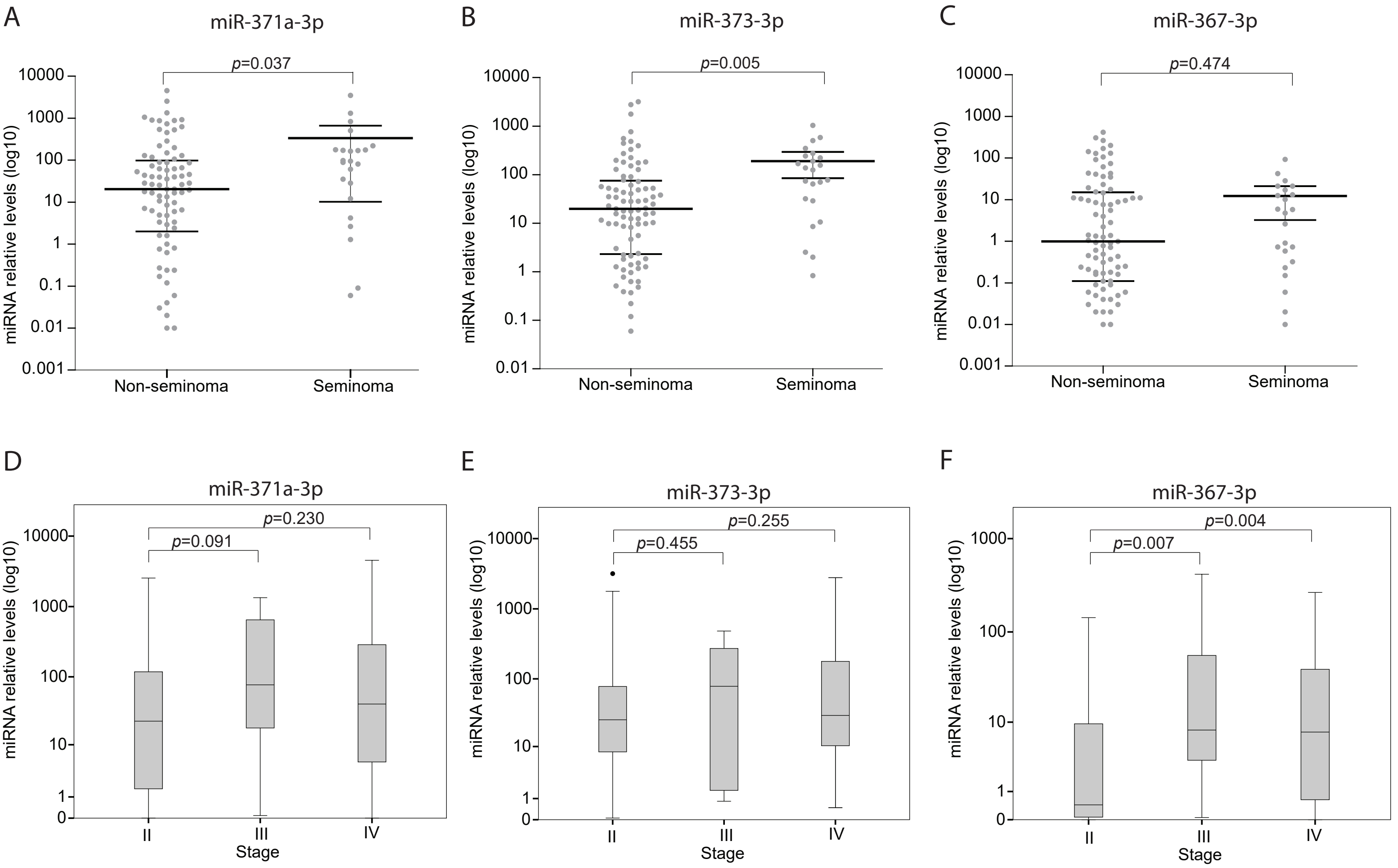

Relative level of miR-371a-3p (A), miR-373-3p (B) and miR-367-3p (C) before start of chemotherapy in cohort 1 and 2 combined comparing non-seminoma (n=85) and seminoma (n=23) tumors. Relative level of miR-371a-3p (D), miR-373-3p (E) and miR-367-3p (F) before start of chemotherapy in cohort 1 and 2 combined comparing stage **II** (n=66), stage **III** (n=12) and stage IV (n=31) tumors. p denotes Mann Whitney U test significance.

Supplementary Figure 3

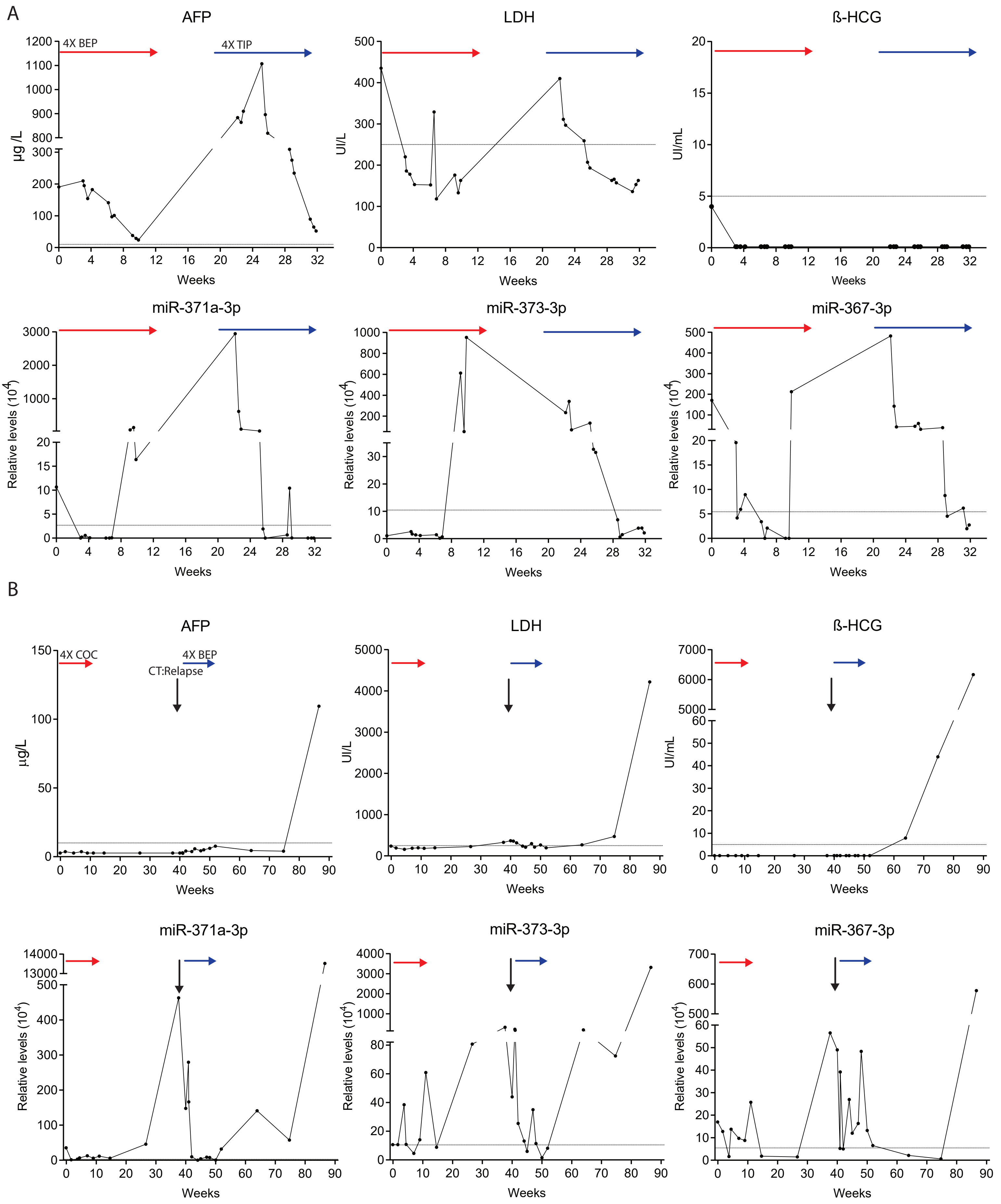

Supplementary Figure 3

Levels of serum markers AFP (µg/L), LDH (UI/L) ß-HCG (UI/mL) and relative levels of miR-371a-3p, miR-373-3p and miR-367-3p at start of chemotherapy, during treatment (red and blue arrows) and follow-up of TGCC case 3 (A) and case 4 (B). Red and blue arrows indicate chemotherapy duration 4xBEP, 4xTIP (paclitaxel, ifosfamide and cisplatin), 4xCOC (cyclophosphamide, vincristine and carboplatin containing chemotherapy).
